# Effect of Consumption Heated Oils with or without Dietary Cholesterol on the Development of Atherosclerosis

**DOI:** 10.3390/nu10101527

**Published:** 2018-10-17

**Authors:** Che Anishas Che Idris, Kalyana Sundram, Ahmad Faizal Abdull Razis

**Affiliations:** 1 Malaysian Palm Oil Board, No. 6, Persiaran Institusi, Bandar Baru Bangi, 43000 Kajang, Selangor, Malaysia; anis@mpob.gov.my; 2 Malaysian Palm Oil Council, 2nd Floor, Wisma Sawit, Lot 6, SS6, Jalan Perbandaran, 47301 Kelana Jaya, Selangor, Malaysia; kalyana@mpoc.org.my; 3 Laboratory of Molecular Biomedicine, Institute of Bioscience, Universiti Putra Malaysia, 43400 UPM Serdang, Selangor, Malaysia; 4 Laboratory of Food Safety and Food Integrity, Institute of Tropical Agriculture and Food Security, Universiti Putra Malaysia, 43400 UPM Serdang, Selangor, Malaysia; 5 Faculty of Food Science and Technology, Universiti Putra Malaysia, 43400 UPM Serdang, Selangor, Malaysia

**Keywords:** heated fats, cholesterol, atherosclerosis, corn oil, palm olein

## Abstract

Heating oils and fats for a considerable length of time results in chemical reactions, leading to the aggravation of a free radical processes, which ultimately contributes to atherosclerosis. Our study focused on elucidating the effect of feeding heated oils with or without dietary cholesterol on the development of atherosclerosis in rabbits. We heated palm olein and corn oil at 180 °C for 18 h and 9 h per day, respectively, for two consecutive days. Next, 20 male rabbits were divided into four groups and fed the following diet for 12 weeks: (i) heated palm olein (HPO); (ii) HPO with cholesterol (HPOC); (iii) heated corn oil (HCO); and (iv) HCO with cholesterol (HCOC). Plasma total cholesterol (TC) was significantly lower in the HCO group compared to the HCOC group. Atherosclerotic lesion scores for both fatty plaques and fatty streaks were significantly higher in the HCO and HCOC groups as compared to the HPO and HPOC groups. Additionally, fibrous plaque scores were also higher in the HCO and HCOC groups as compared to the HPO and HPOC groups. These results suggest that heated palm oil confers protection against the onset of atherosclerosis compared to heated polyunsaturated oils in a rabbit model.

## 1. Introduction

Edible oils and fats form an indispensable component of our diet. They provide a large portion of our energy needs, supply essential fatty acids, and act as carriers for fat-soluble vitamins. They also impart special flavor, texture, and taste to our food so that we can pamper our taste buds as we bite into our favorite foods. In many parts of the world, including Malaysia, there is a tendency for oils to be used repeatedly in frying and cooking. During the frying process, many chemical changes occur, such as oxidation, polymerization, and hydrolysis. These chemical changes can lead to the production of oxidized products [1]. Several observations suggest that a diet rich in oxidized fat can lead to atherosclerosis [2] and ultimately cardiovascular disease [3]. Atherosclerosis is the condition in which an artery wall thickens as a result of the buildup of fatty materials such as cholesterol [4]. This syndrome usually affects arterial blood vessels and occurs in response to chronic inflammatory processes [4]. Many factors have been incriminated in triggering the inflammatory process [5]. One of these is the accumulation of macrophages and white blood cells promoted by low-density lipoproteins. Inadequate removal of fats and cholesterol from the macrophages results in the formation of fatty streaks which ultimately lead to fibrous plaques [6].

In animal studies, highly oxidized and degraded substances caused adverse biological effects including growth retardation, diarrhea, cellular damage, and even death [7,8,9,10,11]. Studies have also shown that heating edible fats at high temperatures and feeding these to animals increase the development of atherosclerotic lesions [8,12,13,14].

Palm oil is one of the world’s most widely consumed oils. Palm oil contains equal portions of saturated and unsaturated fatty acids [15]. It is excellent for deep-fat frying on a continuous basis as it has a balanced saturated-unsaturated fatty acid composition and good oxidative stability. Palm oil also has a significant level of natural antioxidants and has little tendency to form gums and off-flavors [16,17]. Palm olein was also reported to be a stable frying oil [18,19]. Palm olein not only has a higher stability index, [19,20,21,22] but it also extends the shelf life of fried foods [19,21,22,23,24]. 

The effects of heated palm oil on lipid parameters as well as its ability to produce atherosclerosis have yet to be determined. In light of this, our study focused on the effect of feeding heated palm olein with and without cholesterol on the growth rate, organ functions, lipid parameters, and atherosclerotic lesions in male rabbits.

## 2. Materials and Methods

### 2.1. Animal Model

The ethical approval of the study was obtained from Institutional Animal Care and Use Committee (IACUC), Universiti Putra Malaysia. Twenty male New Zealand white rabbits aged four to five months with body weights of approximately 2 kg were assigned into four groups. The animals were housed individually in stainless steel cages and maintained in a temperature-controlled room (18–23 °C) with a 12 h daylight cycle. 

### 2.2. Animal Diets

Animals were fed *ad libitum* on a semi-purified diet, containing 35% energy from fat for 12 weeks and had free access to their drinking water. The rabbits in Group 1 were fed the heated palm olein diet without added dietary cholesterol (HPO); those in Group 2 were fed the heated corn oil diet without added dietary cholesterol (HCO); those in Group 3 were fed the heated palm olein diet with added dietary cholesterol at 0.10% (*w*/*w*) (HPOC); and those in Group 4 were fed the heated corn oil diet with added dietary cholesterol at 0.10% (*w*/*w*) (HCOC). Diets were formulated according to Table 1.

Only two oils were used in this study: palm olein (Vesawit, Yee Lee Edible Oils Sdn Bhd, Ipoh, Perak, Malaysia) and corn oil (Vecorn, Yee Lee Edible Oils Sdn Bhd, Ipoh, Perak, Malaysia). These oils were heated in an electrically heated open fryer (Frymaster, Shreveport, LA, USA) at 180 °C for a total 18 h and nine hours per day, respectively, for two consecutive days. Both the test oils were analyzed for their polar compounds—according to the method of Diefenbaker and Pocklington [25]—polymer compounds [26], free fatty acids [27], induction period [27] (Table 2), and fatty acid contents [27] (Table 3). It was observed that after the 18 h of heating, the linoleic acid contents of both oils decreased, which could be used to indicate the level of oil deterioration [22,28,29]. Total vitamin E content in the heated oils was measured by high performance liquid chromatography [30]. It was found that the total vitamin E content of the heated corn oil was higher than that of the heated palm olein oil. However, the tocol contents were highest in the heated palm olein (Table 4). 

The body weight gain of rabbits was measured weekly. At the end of the experiment, the rabbits were anaesthetized with a mixture of ketamine (50 mg/kg body wt.) and xylazine (10 mg/kg body wt). A 30 mL of blood was then drawn by heart puncture for biochemical analysis. All animals were then euthanized with sodium pentothal (100 mg/kg). Subsequently, the anterior chest wall was removed, and the abdominal cavity was opened to reveal the aorta and its branches. These vessels were excised as a unit, and loose adventitial tissue was removed using blunt dissection. The aorta was then opened longitudinally and pinned flat on a paraffin wax. Aorta was preserved in 10% formalin solution before staining with Oil Red-O for quantification of the atherosclerotic lesions.

### 2.3. Plasma Lipid Analysis

Plasma lipids with total cholesterol (TC), triglycerides (TG), high-density lipoproteins (HDL-C), and low-density lipoproteins (LDL-C) were analyzed using enzymatic assay kits (Roche Diagnostics GmbH, Mannheim, Germany), as per manufacturer’s protocols, using the Clinical Chemistry Autoanalyser, Roche/Hitachi 902 (Roche Diagnostics, F. Hoffmann-La Roche Ltd., Basel, Switzerland).

### 2.4. Plasma Antioxidant Status

Plasma antioxidant status was measured by the 2,2′-azinobis (3-ethylbenzothiazoline) 6-sulfonic acid radical cation (ABTS^•+^) decolonization assay and ferric reducing ability of plasma (FRAP) assay.

The ability of rabbit plasma to scavenge the ABTS^•+^ was measured using the method of Re et al. [31], as adapted by Balasundram [32]. In short, this assay measures the ability of antioxidants in the plasma, at the absorbance of 734 nm, to scavenge preformed ABTS^•+^ produced using the oxidation of ABTS with potassium persulfate. The ABTS^•+^ scavenging capacity of the plasma is reported in terms of mg/mL Trolox equivalents (mg/mL TE).

The FRAP assay was carried out using the method documented by Firuzi et al. [33], as adapted by Balasundram [32]. The original FRAP assay was developed by Benzie and Strain [34] as a test to measure the ferric reducing ability of plasma—i.e., the ability to reduce a ferric-2,4,6-tripyridyl-*s*-triazine (Fe^3+^-TPTZ) to ferrous-2,4,6-tripyridyl-*s*-triazine (Fe^2+^-TPTZ) using an electron transfer mechanism. This reduction resulted in the production of an intensely blue colored reduced complex, with maximum absorption at 593 nm [34]. The FRAP value of the plasma is reported in terms of mg/mL Trolox equivalents (mg/mL TE).

### 2.5. Aortic Examination

The aorta was stained with Oil Red-O. In addition, quantification of the atherosclerotic lesions was executed with the aid of a computer image analyzing software (iSolutionDT-iMTechnology, Bundang-gu, Seongnam-si, Gyeonggi-do, Korea). Lesions were categorized as follows: (a) Fibrous plaques: Raised nodular lesions, continuous, intense red, white hard visible to naked eyes; (b) Fatty plaques: Raised distinct lesions, intensely stained red; (c) Fatty streaks: Lipid accumulation, stained light red; (d) Lesion-free: Healthy intima.

### 2.6. Statistical Analysis

Statistical analysis was conducted using an SPSS software (version 17). Quantitative data were presented as means ± SD. The statistical significance between means was estimated by a one-way analysis of variance (ANOVA), followed with a least significant difference (LSD) multiple comparisons (protected LSD). Moreover, *p* < 0.05 was considered statistically significant.

## 3. Results

### 3.1. Animal Condition

The final weight changes for the surviving animals are shown in Table 5. Of the four groups, only rabbits fed the HPO diet showed an increase in weight gain; this was statistically significant compared to rabbits given the HPOC, HCO, and HCOC diets. The three other groups showed body weight losses, with the highest loss observed in the HCO group, followed by the HCOC and HPOC groups. The HPOC group showed the lowest body weight loss; this was statistically significant when compared to the HPO, HCO, and HCOC groups. Liver weight was the highest in the HPOC group followed by the HPO, HCOC, and HCO groups. However, the weights of hearts showed no significant differences between the four groups. Furthermore, no significant difference was noted in the alanine transaminase (ALT) levels between the groups (Table 5).

### 3.2. Plasma Lipid Analysis

**Figure 1:**
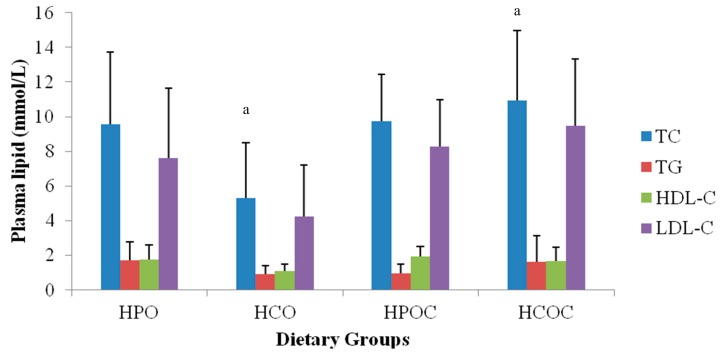
Total cholesterol (TC), triglycerides (TG), high-density lipoproteins (HDL-C), and low-density lipoproteins levels (LDL-C) of rabbits in the four dietary groups. Values are presented in means ± SD and means with similar letters differ significantly (*p* < 0.05). Heated palm olein (HPO); heated palm olein with added dietary cholesterol (HPOC); heated corn oil (HCO); heated corn oil with added dietary cholesterol (HCOC). Similar letters differ significantly.

Figure 1 shows the TC, TG, HDL-C, and LDL-C levels of rabbits in the four dietary groups. TC level was the lowest in the HCO group, but the highest in the HCOC group. This difference was statistically significant between the two groups. As for the TG levels, no significant difference was found between the dietary groups. Plasma LDL-C level was the highest in the HCOC group, followed by the HPOC, HPO, and HCO groups. However, these differences were also not found to be statistically significant.

### 3.3. Plasma Antioxidant Status 

**Figure 2:**
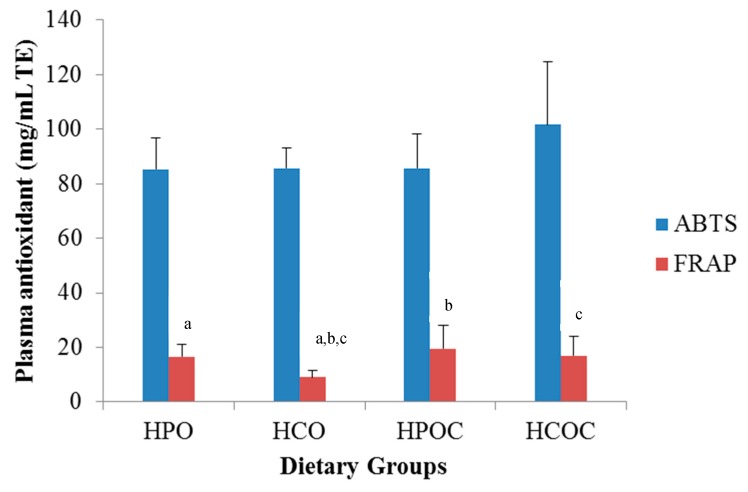
ABTS (2,2′-azinobis (3-ethylbenzothiazoline) 6-sulfonic acid radical cation) and ferric reducing ability of plasma (FRAP) levels of rabbits in the four dietary groups. Values are presented in means ± SD and means with similar letters differ significantly (*p* < 0.05). Similar letters differ significantly.

In order to determine the antioxidant capacity in rabbit plasma (Figure 2), the ABTS assay showed no significant difference between the four dietary groups. On the other hand, the plasma FRAP value was the lowest in the HCO dietary group; this was statistically significant when compared to the HPO, HPOC, and HCOC groups. However, among the HPO, HPOC, and HCOC groups themselves, the differences in FRAP value were not statistically significant.

### 3.4. Aortic Examination

**Figure 3:**
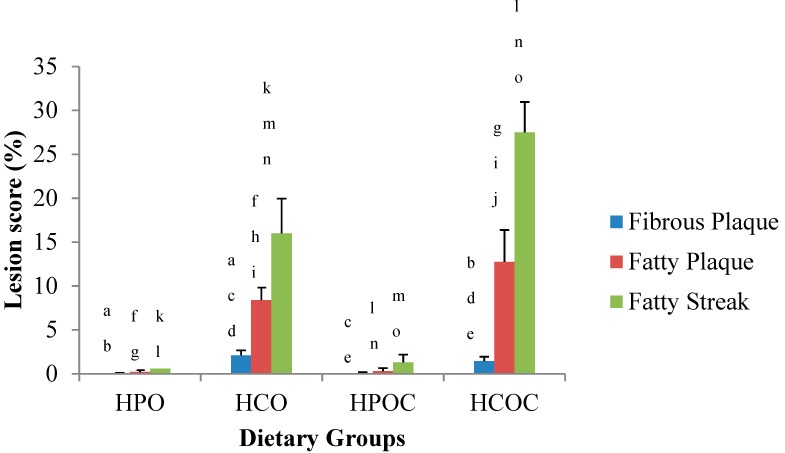
Percentage of fibrous plaques, fatty plaques and fatty streaks in the aortas of rabbits in the four dietary groups. Values are presented in means ± SD and means with similar letters differ significantly (*p* < 0.05). Similar letters differ significantly.

**Figure 4:**
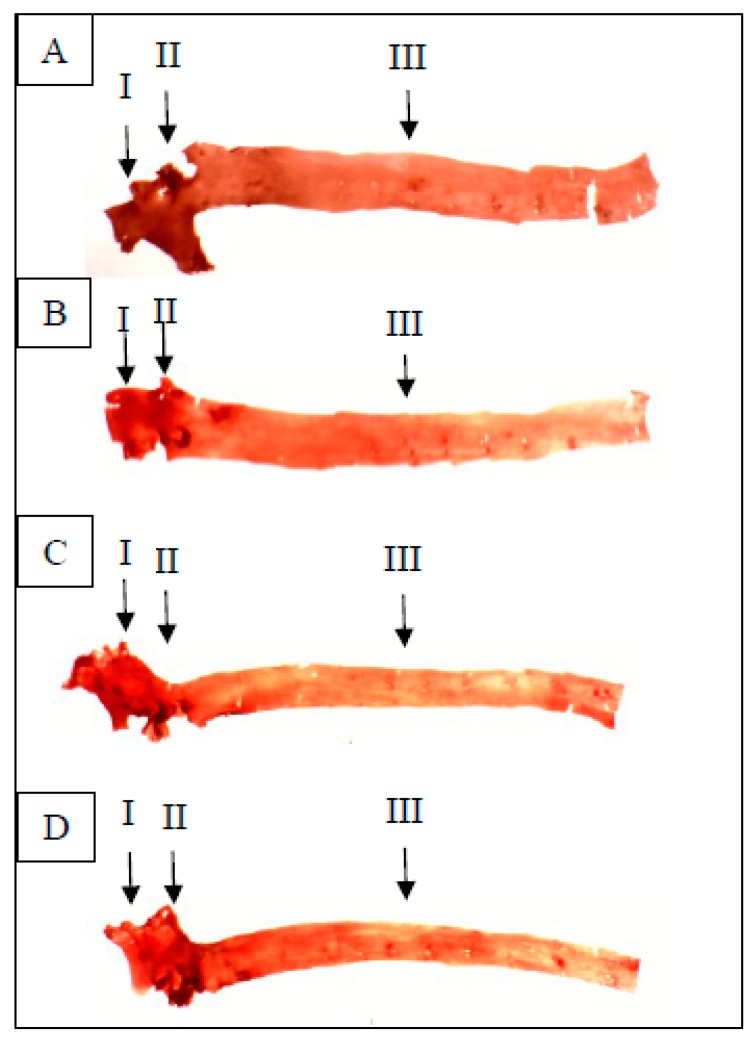
Photographs of aortic lesions in rabbits fed diet with and without added dietary cholesterol in different groups (**A**) HPO, (**B**) HPOC, (**C**) HCO, and (**D**) HCOC. Development of atherosclerosis is shown as (I) fibrous plaques, (II) fatty plaques, and (III) fatty streaks.

In comparing fibrous plaques (Figure 3), the HCO group showed the highest lesion score, followed by the HCOC, HPOC, and HPO groups. Differences in these scores were statistically significant among the groups. When comparing the HPOC and HCOC groups, fibrous plaques seen in the HCOC group was significantly higher than the HPOC group. In comparing fatty plaques and fatty streaks however, the HCOC group showed the highest lesion score followed by the HCO, HPOC, and HPO groups. Differences in these scores were also statistically significant. Figure 4 visualized the development of atherosclerosis in all the rabbits used in this study after being fed the diet with and without added dietary cholesterol for 12 weeks.

## 4. Discussion

The main objective of this study was to evaluate the effect of ingesting heated palm olein with and without added dietary cholesterol, against heated corn oil with and without added dietary cholesterol on the lipid profiles, as well as examining the formation of atheroma in rabbits. Palm olein was chosen for this study because it is one of the most commonly used household and food industry frying oils in the world [35]. Corn oil is also widely used as an all-purpose cooking oil because of its unique flavor attributes. Furthermore, corn oil was said to be more stable than linolenate-containing oils such as soybean and canola [36,37]. 

From the obtained results, only the HPO group showed a positive weight gain as opposed to the HCO, HPOC, and HCOC groups, where decreases in body weights were observed. This was consistent with various studies that reported ingestion of highly oxidized oils and their degraded substances resulted in growth retardation and even death [7,8,38,39]. The observed phenomena could be attributed to the fact that heating oils at high temperatures for long periods of time generally results in oxidation, and that the byproducts of oxidation render the feed less palatable. Of the two oils, corn oil is more unsaturated and more prone to oxidation, thus less palatable, possibly leading to decreases in food intake and body weights. This might explain why rabbits from the HCO, HPOC, and HCOC groups were negative in terms of weight gain. The lower body weights could also be attributed to the intake of long-chain polymers that may cause fat to be less absorbed [39,40,41]. This might then interfere with absorption of other fat-soluble nutrients in the diet [41,42]. Palm olein, on the other hand, has been shown to be more resistant to oxidation. This resistance has been attributed to the higher proportion of tocols in relation to the lower polyunsaturated fatty acid contents [43,44]. The lower degree of oxidation would then make a palm oil-based diet more palatable than a corn oil-based diet, resulting in higher consumption of the palm-oil based diet. Moreover, this may help explain the differences in weight gain between the groups as was observed in the present study.

Long term consumption of oxidized oils has been reported to not only result in growth retardation, but cause liver enlargement [11,45,46,47,48]. In this study, the palm oil-based groups—HPO and HPOC—resulted in heavier liver weights than the corn oil-based groups, HCO and HCOC. No differences in heart weights and ALT levels were observed among the four dietary groups.

TC levels were comparable between the HPO, HPOC, and HCOC groups. However, the HCO group showed a significantly lower level of TC when compared to the other three groups. This finding was consistent with the fact that corn oil, being an unsaturated vegetable oil, did not raise TC. However, the HCOC diet in which dietary cholesterol was added caused TC levels to double. Palm olein naturally contains equal portions of saturated and unsaturated fatty acids. It has been reported that saturated fatty acids have been shown to result in an increased in TC [15], and thus is consistent with the current findings. However, adding cholesterol in the HPOC group did not result in a significant rise in TC. This is postulated to the suppression of endogenous production of cholesterol in the liver by the antioxidant capacity of the oil, resulting in the retardation of further TC increases. Similarly, these observations were made by Idris et al. [49], who found that a 12 week supplementation of palm oil containing vitamin E and phenolics suppressed atherosclerosis in hypercholesterolaemic rabbits, inferring then that the phytochemicals present in the oil suppress atherosclerosis without affecting plasma lipid levels. Other lipid parameters (TG, HDL-C, and LDL-C) showed no significant differences in levels among the four dietary groups.

In this study, plasma antioxidant capacity was measured by two different methods, namely FRAP and ABTS. Results obtained from the ABTS scavenging assay showed no significant differences among the four groups. Using the FRAP analysis, however, the HPO, HPOC, and HCOC groups showed significantly higher antioxidant levels when compared to the HCO group. The different results obtained between the ABTS^•+^ and FRAP methods could be due to the different reaction mechanisms involved. The ABTS^•+^ method is a diammonium salt radical cation scavenging assay, whereas the FRAP method is a ferric reducing power assay [32]. In addition, the antioxidants in plasma consist of complex cascade of enzymatic and non-enzymatic antioxidants, which may have evoked different responses to both of the ABTS^•+^ and FRAP measured [50,51].

In comparing the formation of fibrous plaques, fatty plaques, and fatty streaks between the groups, the corn oil-based diet groups showed a higher percentage in the formation of these lesions when compared to the palm oil-based diet groups. This was in line with the findings by Kritchevsky and Tepper [12], as well as Staprans et al. [13], which showed that heated corn oil was very atherogenic in rabbits. For fibrous plaque formation, the HCO group had the highest percentage of occurrence and this was significant when compared to the HPO, HPOC, and HCOC groups. In the formation of fatty plaques and fatty streaks, however, the HCOC group had the highest percentage of occurrence of these lesions. This finding was expected as other studies have shown that addition of cholesterol in oxidized fats resulted in an overwhelming increase in fatty plaques and fatty streaks in rabbits [14]. However, it was interesting to note that the percentage of all the lesions mentioned was very low in the palm oil-based diet groups, in concordance with the observation made previously by Idris et al. [49]. Even with the addition of cholesterol to palm oil, the increase in the percentage of lesions in the aorta was minimal. In the present study, rabbits in the HPO group did not exhibit significant differences in their plasma lipid profiles (TC, TG, HDL-C and LDL-C) in comparison with HPOC group. This was substantiated by insignificant differences in atherosclerotic lesion scores for both groups, signifying the atheroprotective effects of palm oil due to high antioxidant status based on the FRAP assay.

## 5. Conclusions

To this end, we conclude that palm olein, having a balanced fatty acid composition and an appreciably higher content of fat-soluble antioxidant vitamin E, confers protection against abusive high heat treatments. This was further biologically translated into a reduced tendency towards atherosclerosis as demonstrated in the current rabbit study.

## Figures and Tables

**Table 1:** Experimental diet formulation.

Ingredients	g/kg Diet	
	HPO/HCO	HPOC/HCOC
Casein	250.0	250.0
Corn starch	200.0	200.0
Dextrose	192.5	192.5
Cellulose	152.0	151.0
Mineral Mix	40.0	40.0
Vitamin Mix	10.0	10.0
Choline Bitartrate	2.5	2.5
dl-Methionine	3.0	3.0
Cholesterol	-	1.0
Dietary Fat	150.0	150.0

Heated palm olein (HPO); heated palm olein with added dietary cholesterol (HPOC); heated corn oil (HCO); heated corn oil with added dietary cholesterol (HCOC).

**Table 2:** Effect of heat treatment on the dietary oils used in the experimental diets.

Sample	Polar Compound (%)	Polymer Compound (%)	Free Fatty Acids (%)	Induction Period (110 °C, h)
Unheated Palm Olein	6.79	1.19	0.04	ND
Heated Palm Olein	16.50	1.23	0.76	5.75
Unheated Corn Oil	3.59	1.49	0.07	ND
Heated Corn Oil	15.55	1.72	0.36	1.85

ND: Not determined.

**Table 3:** Percentage distribution of major fatty acids (%) in the unheated and heated oils.

Sample	12:0	14:0	16:0	18:0	18:1	18:2	18:3
Unheated Palm Olein	0.51	0.91	37.88	3.35	46.11	10.86	0.08
Heated Palm Olein	-	0.75	41.06	3.11	45.76	8.97	-
Unheated Corn Oil	-	0.04	7.05	2.76	31.41	61.19	0.11
Heated Corn Oil	-	-	7.64	2.32	28.85	57.47	-

**Table 4:** Tocol contents of unheated and heated palm olein and corn oil.

Sample	α-t (ppm)	α-t3 (ppm)	β-t (ppm)	γ-t (ppm)	γ-t3 (ppm)	δ-t (ppm)	δ-t3 (ppm)	Total (ppm)
Unheated Palm Olein	200.86	204.72	12.19	18.54	329.10	0.76	126.46	892.63
Heated Palm Olein	23.20	15.22	9.43	12.23	30.43	0.47	39.70	139.68
Unheated Corn Oil	309.60	0.00	21.29	98.06	13.45	9.95	0.00	452.35
Heated Corn Oil	95.56	0.00	18.31	69.03	10.81	9.71	0.00	204.42

α-t, alpha-tocopherol; α-t3, alpha-tocotrienol; β-t, beta-tocopherol; γ-t, gamma-tocopherol; γ-t3, gamma-tocotrienol; δ-t, delta-tocopherol; δ-t3, delta-tocotrienol; ppm, part per million.

**Table 5:** Final weight change; liver and heart weights; and ALT levels of rabbits in the four dietary groups.

Dietary Groups	HPO *n* = 5	HCO *n* = 5	HPOC *n* = 5	HCOC *n* = 5
Initial body weight (g)	2054.81 ± 270.70	2000.28 ± 257.98	2071.52 ± 296.20	2079.78 ± 260.42
Final body weight (g)	2157.20 ± 291.71 ^a,b,c^	1681.50 ± 323.02 ^a,d^	2045.26 ± 238.78 ^b,d,e^	1906.98 ± 150.64 ^c,e^
Final weight change (g)	90.27 ± 99.82 ^a,b,c^	−323.14 ± 34.66 ^a,d^	−37.10 ± 86.92 ^b,d,e^	−275.37 ± 138.92 ^c,e^
Liver weight (g)	40.66 ± 8.59 ^a,b^	30.72 ± 3.42 ^a,c^	44.02 ± 4.82 ^c,d^	31.35 ± 5.15 ^b,d^
Heart weight (g)	4.10 ± 0.78	3.24 ± 0.45	3.87 ± 0.52	3.35 ± 0.55
ALT (IU/L)	58.14 ± 48.56	48.99 ± 24.71	37.78 ± 14.62	38.49 ± 28.22

Values are means ± SD. ^a–e^ Values within a horizontal row and with similar superscripts differ at *p* < 0.05. *n* = number of animals; heated palm olein (HPO); heated palm olein with added dietary cholesterol (HPOC); heated corn oil (HCO); heated corn oil with added dietary cholesterol (HCOC); alanine transaminase (ALT).
