# Correction: Che Idris, C.A., et al. Effect of Consumption Heated Oils with or without Dietary Cholesterol on the Development of Atherosclerosis. *Nutrients* 2018, 10, 1527

**DOI:** 10.3390/nu10121839

**Published:** 2018-11-30

**Authors:** Che Anishas Che Idris, Kalyana Sundram, Ahmad Faizal Abdull Razis

**Affiliations:** 1 Malaysian Palm Oil Board, No. 6, Persiaran Institusi, Bandar Baru Bangi, 43000 Kajang, Selangor, Malaysia; anis@mpob.gov.my; 2 Malaysian Palm Oil Council, 2nd Floor, Wisma Sawit, Lot 6, SS6, Jalan Perbandaran, 47301 Kelana Jaya, Selangor, Malaysia; kalyana@mpoc.org.my; 3 Laboratory of Molecular Biomedicine, Institute of Bioscience, Universiti Putra Malaysia, 43400 UPM Serdang, Selangor, Malaysia; 4 Laboratory of Food Safety and Food Integrity, Institute of Tropical Agriculture and Food Security, Universiti Putra Malaysia, 43400 UPM Serdang, Selangor, Malaysia; 5 Faculty of Food Science and Technology, Universiti Putra Malaysia, 43400 UPM Serdang, Selangor, Malaysia

The authors wish to make the following change to their paper (Che Idris et al. 2018 [1]). The changes concern the declaration of Conflicts of Interest. The correct Conflicts of Interest are included in the section below. The authors would like to apologize for any inconvenience caused to the readers by this change. The change does not affect the results and we apologize for the oversight. The manuscript will be updated and the original will remain online on the article webpage.

**Conflicts of Interest:** C.A.C.I. is currently working for the Malaysian Palm Oil Board, K.S. is currently from the Malaysian Palm Oil Council which previously served the Malaysian Palm Oil Board, and A.F.A.R. did his research internship at the Malaysian Palm Oil Board.
